# Characterization of *Pseudomonas aeruginosa* isolates from patients with endophthalmitis using conventional microbiologic techniques and whole genome sequencing

**DOI:** 10.1186/s12348-020-00216-0

**Published:** 2020-09-28

**Authors:** Jesse D. Sengillo, Jacob Duker, Maribel Hernandez, Jorge Maestre, Daniela Reyes-Capo, Annika Patel, Arjun Watane, Nimesh A. Patel, Nicolas A. Yannuzzi, Darlene Miller, Harry W. Flynn

**Affiliations:** Department of Ophthalmology, Bascom Palmer Eye Institute, University of Miami Miller School of Medicine, 900 NW 17th Street, Miami, FL 33136 USA

**Keywords:** *Pseudomonas aeruginosa*, Whole genome sequencing, Endophthalmitis

## Abstract

**Purpose:**

To demonstrate antibiotic susceptibility and genomic virulence factor profiles of *Pseudomonas aeruginosa* isolates from patients with culture-confirmed endophthalmitis.

**Methods:**

Clinical isolates from patients diagnosed with pseudomonas endophthalmitis were included. Laboratory antibiotic susceptibility testing and whole genome sequencing was performed on all isolates.

**Results:**

In the current study, 8 patients had vitreous culture-confirmed endophthalmitis due to *P. aeruginosa*. All isolates were multi-drug resistant but sensitive to ceftazidime and each fluoroquinolone tested. Whole genome sequencing revealed a total of 179 unique genes. The most common type of virulence genes included those involved in adherence and the secretion system. Seven of 8 (88%) isolates were of the cytoinvasive phenotype (*exoST*) and no isolates contained *exoU*.

**Conclusions:**

*P. aeruginosa* associated endophthalmitis is often multi-drug resistant and demonstrates a variety of virulence factors with those involved in adherence and the secretion system being the most common.

## Background

Infectious endophthalmitis is a severe sight-threatening entity that can occur post-operatively, following trauma, or coincident with systemic infection. In the Endophthalmitis Vitrectomy Study (EVS) study, 36% of patients with endophthalmitis failed to achieve better than 20/100 visual acuity at 9 to 12 months [1]. Though just 4.1% of isolates in the EVS study were gram-negative organisms, other studies suggest a higher incidence that ranges from 10 to 24% [2–8]. Among gram-negative endophthalmitis cases, *Pseudomonas aeruginosa* is the most commonly isolated organism [3, 9–12] and is associated with a more fulminant clinical course and higher evisceration/enucleation rate compared to its gram-positive counterparts [13–15]. In fact, a recent prospective study by Stevenson et al. reported 30% of patients with gram negative endophthalmitis requiring evisceration or enucleation [8].

In addition to the growing prevalence of multi-drug resistant strains, the virulent nature of pseudomonas is often ascribed to factors expressed by its bacterial DNA [16]. These virulence factors contribute to its ability to induce rapid ocular tissue necrosis [6]. Comparative studies correlating these genotypes with clinical features observed in host tissue increase our understanding of pseudomonas pathogenicity [17]. However, there is a paucity of studies which have sought to identify virulence factors implicated in cohorts of pseudomonas keratitis and endophthalmitis [17, 18]. In the current study, whole genome sequencing (WGS) was performed on isolates to identify associated virulence factors in culture-confirmed pseudomonas endophthalmitis.

## Methods

The current study was approved by the Institutional Review Board of the University of Miami School of Medicine Medical Sciences Subcommittee for the Protection of Human Subjects, and was performed in accordance with the ethical standards as laid down in the 1964 Declaration of Helsinki and later amendments. Clinical and microbiology records were retrospectively reviewed for patients who were evaluated at Bascom Palmer Eye Institute and diagnosed with vitreous culture-confirmed endophthalmitis due to *P. aeruginosa*. Antibiotic susceptibility profiles were identified using standard microbiologic protocols via an automated system, including the VITEK (Automatic Microbial System; Biomerieux Vitek, Hazelwood, Missouri, USA) which provided ‘breakpoint’ MICs (minimal inhibitory concentration values) based on the micro dilution method, the E test (A.B. Biodisk, NA; Remel, Lenexa, Kansas, USA), or disk diffusion (antibiotic-impregnated paper disks; Becton Dickinson, Cockeysville, MD). Whole genome sequencing was performed by COSMOS ID (Rockville, MD) using Illumina and Ion Torrent platforms.

## Results

The current study includes 8 patients diagnosed with vitreous culture-confirmed *P. aeruginosa* endophthalmitis at Bascom Palmer Eye Institute. Clinical data is presented in Table 1. The average age was 74 years (range: 53–84). Four of 8 patients (50%) were men. Endophthalmitis was diagnosed based on clinical findings and confirmed with vitreous cultures obtained via tap or pars plana vitrectomy. Three of 8 cases (38%) were in the setting of recent ocular surgery, which included post-phacoemulsification (*n* = 2) and post-corneal transplant (*n* = 1). Three cases occurred in the setting of corneal ulceration, with one patient exhibiting scleral extension of infection (P1). One case followed an open globe injury with a retained intraocular foreign body. Multiple organisms grew from vitreous cultures in two patients, namely *Staphylococcus aureus* (P5) and *Staphylococcus hominis* (P7).

**Table 1:** Clinical characteristics of of patients with endophthalmitis due to *Pseudomonas aeruginosa*

Patient	Sex	Age (yrs)	Clinical setting	Concurrent corneal ulcer	Time after surgery	Additional isolates from vitreous
1	F	81	Sclerokeratitis	Yes	–	–
2	F	69	Post phacoemulsification	No	5 days	–
3	F	80	UK	UK	UK	–
4	M	71	Corneal ulcer	Yes	–	–
5	M	84	Postoperative phacoemulsification	No	5 days	Staphylococcus aureous
6	F	80	Post corneal transplant	No	8 days	–
7	M	78	Corneal ulcer	Yes	–	Staphylococcus hominis
8	M	53	Globe rupture with IOFB	No	–	–

Clinical characteristics of patients with *P. aeruginosa* isolated from vitreous cultures in this cohort

*Abbreviations*: *UK* unknown, – not applicable, *IOFB* intraocular foreign body. Detailed clinical records were not available for P3

Average follow-up time for patients in this cohort was 2 years. Clinical presentation, treatment strategy, and visual outcomes are detailed in Table 2. Baseline visual acuity prior to diagnosis of endophthalmitis was not available. Vision at presentation was hand motions or light perception for all patients with documented visual acuity (7 of 8 patients). Final visual outcome ranged from 20/400 to no light perception, with 2 patients ultimately requiring enucleation. Intravitreal tap and injection of antibiotics were used as initial treatment for 5 of 8 cases, while pars plana vitrectomy (PPV) with intraoperative intravitreal injections was performed initially in 2 of 8 cases. For P1 and P4, data was not available regarding initial type of intravitreal antibiotic and for P3, initial treatment choice (PPV vs intravitreal injections) was not indicated in the medical record.

**Table 2:** Presentation, treatment strategies, and outcomes of patients with endophthalmitis caused by *Pseudomonas aeruginosa*

No.	Baseline VA	Initial VA	Initial Tx	Initial IVTI	Additional Tx (Days after presentation)	Additional IVTI (Days after presentation)	Last VA	Follow-up time
1	UK	LP	T + I	Unknown	PPV (1)	Cfx (2) Ctz (3)	NLP	2 years
2	UK	LP	PPV	Vanc + Ctz (intra-op)	Enucleation	–	Enucleation	7 years
3	UK	UK	UK	UK	UK	UK	UK	UK
4	UK	HM	T + I	UK	Enucleation (6)	–	Enucleation	8 months
5	UK	LP	T + I	Vanc + Ctz + Dex	PPV (4)	Vanc + Dex (4) Ctz (8)	CF	3 months
6	UK	LP	T + I	Vanc + Ctz	PPV (4)	Ctz (2 & 8) Ctz intra-op (4)	LP	2 days
7	UK	LP	T + I	Ctz + Vanc	–	Vanc + Dex (2) Vanc + Ctz + Dex (5)	LP	4 years
8	UK	HM	Globe repair PPV	Vanc + Ctz + Vcz (intra-op)	Retinal detachment repair (4 months)	–	20/400	2 years

Presentation, treatment strategies, and outcomes of patients with endophthalmitis caused by *Pseudomonas aeruginosa* in this cohort

*Abbreviations*: *VA* visual acuity, *NLP* no light perception, *LP* light perception, *HM* hand motions, *UK* unknown, *Tx* treatment, *IVTI* intravitreal injection, *T + I* vitreous tap and intravitreal injection, *PPV* pars plana vitrectomy, *Vanc* vancomycin, *Ctz* ceftazidime, *Cfx* cefuroxime, *Dex* dexamethasone, *Vcz* voriconazole, – not applicable

*P. aeruginosa* was identified from the vitreous sample of each patient. Antibiotic sensitivities are summarized in Table 3 and minimum inhibitory concentration values are reported in Supplemental Table 1. All isolates were multi-drug resistant with similar resistance profiles. Sensitivity to ceftazidime was identified across the entire cohort. Vancomycin resistance was not specifically tested, but all isolates were sensitive to the fourth generation cephalosporin, cefepime. Sensitivity to all tested aminoglycosides and fluoroquinolones was seen in all isolates in this cohort, including the newest fluoroquinolone, delafloxacin. Resistance to first- and second- generation cephalosporins, cefazolin and cefoxitin respectively, ampicillin, ampicillin/sulbactam, ceftriaxone, and nitrofurantoin across all isolates was observed. Seven of 8 (88%) isolates were resistant to sulfamethoxazole/trimethoprim.

**Table 3:** Antibiotic sensitivities of *Pseudomonas aeruginosa* vitreous isolates

	Vitreous isolate sensitivities							
Antibiotic	1	2	3	4	5	6	7	8
Ampicillin	R	R	R	R	R	R	R	R
Amp/Sulbactam	R	R	R	R	R	R	R	R
Piper/Tazo	S	–	S	S	S	S	S	S
Ticar/Clav	–	S	–	–	–	–	–	–
Cefazolin	R	R	R	R	R	R	R	R
Cefoxitin	R	R	R	R	R	R	R	R
Ceftazidime	S	S	S	S	S	S	S	S
Ceftriaxone	R	R	R	R	R	R	R	R
Cefepime	S	S	S	S	S	S	S	S
Imipenem	S	S	S	S	S	S	S	S
Meropenem	S	–	S	S	S	S	S	S
Levofloxacin	S	S	S	S	S	S	S	S
Ciprofloxacin	S	S	S	S	S	S	S	S
Moxifloxacin	S	S	S	S	S	S	S	S
Delafloxacin	S	S	S	S	S	S	S	S
Gentamicin	S	S	S	S	S	S	S	S
Amikacin	S	S	S	S	S	S	S	S
Tobramycin	S	S	S	S	S	S	S	S
Trimeth/Sulfa	R	R	R	R	R	R	S	R
Nitrofurantoin	R	R	R	R	R	R	R	R

Antibiotic sensitivities of *Pseudomonas aeruginosa* vitreous isolates from patients with endophthalmitis. Sensitivities were calculated with the VITEK-2 automated system, E-test, or disk diffusion testing. S, sensitive; R, resistant; —, not tested

*Abbreviations*: *Amp/Sulbactam* Ampicillin/Sulbactam, *Piper/Tazo* Piperacillin/Tazobactam, *Ticar/Clav* Ticarcillin/Clavulanic acid, *Trimeth/Sulfa* Trimethoprim/Sulfamethoxazole

All isolates in this study underwent WGS to identify known virulence factors. A total of 1087 virulence genes (179 unique) were identified, with an average of 136 genes per isolate. The number of genes for a particular virulence class in the cohort and per isolate is summarized in Table 4. Genes involved in adherence were among the most prevalent (69 unique genes), followed by those involved in secretion systems (*n* = 47), anti-phagocytosis (*n* = 21), and iron uptake (*n* = 13). Regarding genes implicated in the type III secretion system (T3SS), all isolates harbored *exoT* and 7 of 8 isolates (88%) were found to have *exoS*, while no isolates contained *exoU*. Isolates were identical with regards to virulence genes known to be involved in protease functions, regulation, biosurfactant, and pigmentation. Among the remaining virulence classes there was a high level of homogeneity with only 22% of genes being represented in less than half of the isolates. All virulence genes identified in this study are listed in Supplemental Table 2.

**Table 4:** Virulence gene classes identified in cohort

	Total genes		Unique Genes	
Virulence class	No. in cohort	Average per isolate	No. in cohort	Class represented in all isolates (yes/no)
Adherence	410	51.3	69	Yes
Secretion System	258	32.3	47	Yes
Anti-phagocytosis	152	19.0	21	Yes
Iron uptake	61	7.6	13	Yes
Motility	53	6.6	8	Yes
Toxin	38	4.8	5	Yes
Regulation	32	4.0	4	Yes
Protease	24	3.0	3	Yes
Biosurfactant	16	2.0	2	Yes
Pigment	16	2.0	2	Yes
Exoenzyme	8	1	1	Yes
Endotoxin	2	1	1	No (2/8 isolates)
Other	17	2.1	3	Yes
Total	1087	136	179	

Genes identified among *Pseudomonas aeruginosa* isolates from vitreous cultures of patients with endophthalmitis using whole genome sequencing

## Discussion

The World Health Organization declared *P. aeruginosa* as a critical priority amongst current pathogens urgently in need of new effective antibiotics [19, 20]. A recent study in South India showed a rising resistance to fluoroquinolones, amikacin, and ceftazidime in pseudomonas endophthalmitis, particularly in post-surgical cases [21]. Comparable retrospective studies performed in the United States have not observed a similar increase in resistance yet [14]. In the present study, cultures were performed between 2011 and 2018, with the majority of patients (5 of 8) presenting in 2015 or later. All isolates were sensitive to ceftazidime, which is generally used as a first line intravitreal antibiotic along with vancomycin at this institution. Interestingly, isolates were pan-sensitive to fluoroquinolones tested, including delafloxacin, a newly registered fluoroquinolone not currently used in the treatment of ocular infections [22]. This resistance profile suggests there may be significant geographic variation, which may be better studied in larger cohorts. Specialists should consider local resistance patterns when determining treatment.

*P. aeruginosa* expresses many virulence factors that contribute to its pathogenicity in ocular tissue [18]. In the current study, the most represented virulence factors were those involved in adherence with a total of 69 unique genes of this class identified. Proteins expressed by these genes likely allow pseudomonas to adhere to various intraocular structures. Unique to post operative endophthalmitis, intraocular foreign material, such as a lens implant can serve as a surface for bacteria to attach, and potentially as a nidus for bacteria to grow within a biofilm. Some of the most cited genes implicated in biofilm production include *algD, rhlR, rpoS, and rpoN,* the latter two providing anti-phagocytotic capability against host defenses [23–25]. All isolates contained these genes with the exception of *rpoN*. Notably not identified by whole genome sequencing in this cohort were *pslD*, *pelF*, and *gacS*, genes previously determined to be common amongst biofilm-producing pseudomonas isolates [23, 25]. Genes involved in c-di-GMP regulation are also well-established in the pathophysiology of biofilm production, but were not surveyed in this study, including *siaD, nbdA, dipA, cdrA, PA4781, or PA4108* [26]. However, identifying these genes and performing functional assays of clinical isolates in future studies could provide a new avenue for understanding biofilm potential and prevalence in endophthalmitis isolates.

In the current study, all isolates harbored the gene encoding exoenzyme T (*exoT).* All but one isolate harbored both *exoS* and *exoY*, while *exoU* was not identified. These genes express toxins involved in the T3SS. T3SS is a complex of cellular structures and proteins that allows gram negative organisms to inject proteins directly into host cells, thereby circumventing extracellular obstacles [27]. Exoenzymes are the effector proteins of this system [18]. Generally, strains possessing *exoS* and *exoT* are invasive while strains possessing *exoU* are observed to be cytotoxic in nature. Consistent with previous studies, *exoS* and *exoU* were mutually exclusive, as all strains that contained *exoS,* did not have *exoU* [28–30]. Interestingly, previous reports suggest a predominance of *exoU* strains in isolates from keratitis [28], though even in endophthalmitis arising in the setting of a corneal ulcer in this cohort, this cytotoxic genotype was not present. This may suggest that pseudomonas with a predilection for endophthalmitis may be more commonly of the cytoinvasive type (*exoST*). Lastly, the *exoU* genotype is associated with fluoroquinolone resistance in previously studied clinical isolates [31, 32]. The putative correlation of pan-sensitivity to fluoroquinolones and lack of *exoU* genotype in this cohort should be confirmed in larger studies.

To the authors’ knowledge, this is the first report in which WGS was used to characterize pseudomonas isolates from vitreous cultures of patients with endophthalmitis. As gene expression assays and functional tests were not performed, the authors cannot guarantee that the presence of a virulence gene indicates a role for the respective factor in pseudomonas pathogenesis. Additionally, variability in the clinical context of infection and relative genetic homogeneity between isolates limits the clinical correlations that can be made. Specifically, in this cohort endophthalmitis occurred in the setting of ocular surface disease for 3 patients, post-surgically for 3 patients, and in the setting of trauma for one patient. It is likely that the setting in which infection occurs and the interplay of pseudomonas with host factors play a large role in disease pathogenesis as well [33]. Elucidating this interaction will require a larger cohort. Lastly, in the present study, the genotypic spectrum identified from pseudomonas endophthalmitis isolates were not compared to environmental strains. Such comparisons in future studies would help confirm if particular virulence factors are more common in the setting of endophthalmitis.

## Conclusions

In the current study of *P. aeruginosa* isolates from patients with endophthalmitis, infection occurred in a variety of clinical settings and all organisms were multi-drug resistant. Using whole genome sequencing, many unique virulence factors were identified, with those involved in bacterial adherence, the secretion system, and anti-phagocytosis being the most common. This investigation increases current understanding of the pathogenesis of *P. aeruginosa* endophthalmitis and emphasizes the need for further investigation of these mechanisms.

## Supplementary information

**Additional file 1 **: **Table S1.** Antibiotic sensitivity values for individual isolates.

**Additional file 2 **: **Table S2.** Virulence factor genes identified in cohort.

## Data Availability

The datasets generated and/or analyzed during the current study are available from the corresponding author on reasonable request.

## Abbreviations

*P. aeruginosa*: *Pseudomonas aeruginosa*
EVS: Endophthalmitis Vitrectomy Study
WGS: Whole genome sequencing
PPV: Pars plana vitrectomy
T3SS: Type III secretion system
