# Supplementary material for: Characterization of Pseudomonas aeruginosa isolates from patients with endophthalmitis using conventional microbiologic techniques and whole genome sequencing
Source: J Ophthalmic Inflamm Infect. 2020 Sep 28;10:25. doi: 10.1186/s12348-020-00216-0 (PMC7520479; doi:10.1186/s12348-020-00216-0)
Supplement: Supplementary file 1 — Additional file 1 : Table S1. Antibiotic sensitivity values for individual isolates. [file 12348_2020_216_MOESM1_ESM.docx]

**Supplemental Table 1.** Antibiotic sensitivity values for individual isolates

|  |  | **Vitreous Isolate Sensitivities** | | | | | | | |
| --- | --- | --- | --- | --- | --- | --- | --- | --- | --- |
| **Antibiotic** | **Unit** | **1** | **2** | **3** | **4** | **5** | **6** | **7** | **8** |
| Ampicillin | (μg/ml) | >= 32 R | >=32 R | >=32 R | >=32 R | >=32 R | >=32 R | >=32 R | >=32 R |
| Amp/Sulbactam | (μg/ml) | >=32 R | >=32 R | >=32 R | >=32 R | >=32 R | >= 32 R | >=32 R | >=32 R |
| Piper/Tazo | (μg/ml) | 8 S | —— | 8 S | 8 S | <= 4 S | 8 S | 8 S | 8 S |
| Ticar/Clav | (μg/ml) | —— | 32 S | —— | —— | —— | —— | —— | —— |
| Cefazolin | (μg/ml) | >=64 R | >=64 R | >=64 R | >=64 R | >=64 R | >=64 R | >=64 R | >=64 R |
| Cefoxitin | (μg/ml) | >=64 R | >=64 R | >=64 R | >=64 R | >=64 R | >=64 R | >=64 R | >=64 R |
| Ceftazidime | (μg/ml)^†^ | 0.75 S | 1.00 S | 0.75 S | 0.75 S | 1.00 | 1.50 | 0.75 | 1.00 |
| Ceftriaxone | (μg/ml) | >=64 R | 32 R | 16 R | 32 R | 16 R | 32 R | 32 R | 32 R |
| Cefepime | (μg/ml) | 2 S | 2 S | <=1 S | 2 S | <=1 S | 2 S | 4 S | <=1 S |
| Imipenum | (μg/ml)^†^ | 2.00 S | 0.38 S | 1.00 S | 1.50 S | 1.50 | 2.00 | 1.50 | 1.50 |
| Meropenem | (μg/ml) | <=0.25 S | —— | <=0.25 S | <=0.25 S | 0.5 S | <=0.25 S | 0.5 S | <=0.25 S |
| Levofloxacin | (μg/ml)^†^ | 1.00 S | 0.75 S | 0.38 S | 0.75 S | 0.38 | 0.50 | 0.75 | 0.75 |
| Ciprofloxacin | (μg/ml)^†^ | 0.125 S | 0.13 S | 0.094 S | 0.125 S | 0.06 | 0.13 | 0.13 | 0.13 |
| Moxifloxacin | (μg/ml)^†^ | 1.50 S | 1.00 S | 0.50 S | 1.00 S | 0.50 | 0.75 | 1.00 | 1.00 |
| Delafloxacin | (mm)^‡^ | 22 S | 23 S | 28 S | 23 S | 28 S | 24 S | 22 S | 22 S |
| Gentamicin | (μg/ml) | <=1 S | 4 S | <=1 S | <=1 S | <=1 S | <=1 S | <=1 S | <=1 S |
| Amikacin | (μg/ml) | <=2 S | 4 S | <=2 S | <=2 S | <=2 S | <=2 S | <=2 S | <=2 S |
| Tobramycin | (μg/ml) | <=1 S | <=1 S | <=1 S | <=1 S | <=1 S | <=1 S | <=1 S | <=1 S |
| Trimeth/Sulfa | (μg/ml) | 160 R | >=320 R | 160 R | >=320 R | 80 R | 160 R | 8 S | 80 R |
| Nitrofurantoin | (μg/ml) | >=512 R | >512 R | >=512 R | >=512 R | >=512 R | >=512 R | >=512 R | >=512 R |

Antibiotic sensitivity values of Pseudomonas aeruginosa vitreous isolates from patients with endophthalmitis. Sensitivities were calculated with the VITEK-2 automated system unless noted otherwise as follows: †, E-test and ‡ , disk diffusion testing. Abbreviations: Amp/Sulbactam, Ampicillin/Sulbactam; Piper/Tazo, Piperacillin/Tazobactam; Ticar/Clav, Ticarcillin/Clavulanic acid; Trimeth/Sulfa, Trimethoprim/Sulfamethoxazole.
