# Supplementary material for: Characterization of Pseudomonas aeruginosa isolates from patients with endophthalmitis using conventional microbiologic techniques and whole genome sequencing
Source: J Ophthalmic Inflamm Infect. 2020 Sep 28;10:25. doi: 10.1186/s12348-020-00216-0 (PMC7520479; doi:10.1186/s12348-020-00216-0)
Supplement: Supplementary file 2 — Additional file 2 : Table S2. Virulence factor genes identified in cohort. [file 12348_2020_216_MOESM2_ESM.docx]

**Supplemental Table 2.** Virulence factor genes identified in cohort.

| **Virulence Class** | **Genes common to all isolates** | **Other genes identified** |
| --- | --- | --- |
| Adherence | *chpD, chpE, fimT, fimV, fimX, fleQ, fleR, flgC, flgD, flgE, flgF, flgH, flgI, flgJ, flhA, flhB, flhF, fliC, fliE, fliF, fliG, fliJ, fliM, fliN, pilB, pilD, pilF, pilG, pilH, pilI, pilJ, pilK, pilQ, pilR, pilS, pilT, pilU, pilY1, waaA* | *chpB, chpC, fimU, fleN, flgK, flgL, fliD, fliH, fliI, fliO, fliP, fliQ, fliR, pilA, pilC, pilE, pilM, pilN, pilO, pilP, pilV, pilW, pilX, pilY2, waaC, waaF, waaG, waaP, wzy, wzz* |
| Secretion System | *clpV1, exsA, exsB, exsC, exsD, exsE, flgM, flgN, fliK, hcp1, icmF1, pcrG, pcrH, pcrV, popD, ppkA, pscF, vgrG1, xcpP, xcpT, xcpZ* | *fliT, pcr1, pcr3, pcr4, pcrR, pscB, pscD, pscE, pscG, pscH, pscI, pscJ, pscK, pscL, pscO, pscQ, pscR, pscS, pscT, xcpQ, xcpR, xcpS, xcpU, xcpV, xcpW, xcpY* |
| Anti-phagocytosis | *alg8, alg44, algA, algB, algD, algF, algG, algJ, algL, algI, algQ, algR, algU, algX, algZ, hasA, mucA* | *algE, algK, mucB, mucC* |
| Iron uptake | *fptA, pchR, pvdA, pvdE, pvdS* | *fpvA, pchA, pchB, pchC, pchD, pchG, pchH, pchI* |
| Motility | *flgA, flgB, flgG, fliL, motA* | *flaG, fliA, fliS* |
| Toxin | *exoT, plcH, toxA* | *exoS, exoY* |
| Regulation | *lasR, lasT, rhlR, rpoS* | *——* |
| Protease | *aprA, lasA, lasB* | *——* |
| Biosurfactant | *rhlA, rhlB* | *——* |
| Pigment | *phzM, phzS* | *——* |
| Exoenzyme | *plcB* | *——* |
| Endotoxin | *——* | *wbpB* |
| Other | *motB, orfX* | *wzx* |

Genes identified by whole genome sequencing. Genes which were common to all isolates are listed in the second column. Genes identified in the cohort, but not amongst all isolates are listed in the third column.
